# The Efficacy of Psychoeducational Family Intervention for Major Depression: Study Protocol of a Randomized Controlled Trial

**DOI:** 10.3390/brainsci13081199

**Published:** 2023-08-13

**Authors:** Claudia Toni, Mario Luciano, Eleonora Arsenio, Alessia Boiano, Emilia Corvino, Bianca Della Rocca, Maria Vita Lapadula, Lucia Tretola, Gaia Sampogna, Andrea Fiorillo

**Affiliations:** Department of Psychiatry, University of Campania “Luigi Vanvitelli”, 80138 Naples, Italy; claudia.toni@studenti.unicampania.it (C.T.); eleonora.arsenio@studenti.unicampania.it (E.A.); alessia.boiano@unicampania.it (A.B.); emilia.corvino1@studenti.unicampania.it (E.C.); bianca.dellarocca@unicampania.it (B.D.R.); mariavita.lapadula@studenti.unicampania.it (M.V.L.); lucia.tretola@unicampania.it (L.T.); gaiasampogna@gmail.com (G.S.); andrea.fiorillo@unicampania.it (A.F.)

**Keywords:** family psychoeducation, major depression, caregiver, family burden, relapse rates, depressive symptoms, social contacts

## Abstract

This study aims to assess the efficacy of a psychoeducational family intervention (PFI) to reduce the severity of depressive symptoms and to improve psychosocial functioning and to increase social contacts in a sample of patients with major depressive disorder (MDD). The degree to which PFI will reduce patients’ relapses, hospitalizations, and self-stigmatization and will improve their quality of life will also be assessed. Other secondary outcomes include the improvement of relatives’ coping strategies, family burden, expressed emotions and quality of life. This non-profit, unfunded, national, multicentric randomized controlled trial with blinded outcome assessments will be carried out in 24 Italian university outpatient units. Families will be assessed at baseline and at 6, 12, and 24 months post-randomization. Our working hypothesis is that the PFIs will reduce the patients’ severity of depressive symptoms, their relapses, and their hospitalizations, and that they will improve their psychosocial functioning and quality of life. We expect these results to be maintained after 12 and 24 months, albeit with a reduction in magnitude. The sample will consist of 384 patients randomized at a 1:1 ratio and stratified according to center, age, gender, and educational level.

## 1. Introduction

Major depressive disorder (MDD) is the most common mental disorder, with prevalence rates ranging from 7.2% to 10.8% in the general population [1]. It is a highly recurrent disorder [2], with approximately 50% of patients experiencing a relapse after a first episode, 70% after a second episode, and 90% after a third one [3].

According to the World Health Organization, MDD is among the top 10 causes of disability worldwide, and it is associated with high personal and societal costs and a significant reduction in the quality of life of both patients and their relatives [4,5].

Caring for a patient with MDD can be highly demanding and cause significant distress in family members, as is evidenced by the high rates of divorce and the frequent financial difficulties found among patients with MDD and their family members [6]. Balkaran et al. report that the levels of subjective and objective burden experienced by relatives of patients with depression are significantly higher than those reported by the families of patients with other mental disorders, including schizophrenia and bipolar disorder [7]. Relatives of patients with MDD often report financial difficulties, reduced social activity, concerns about stigma and prejudice toward people with depression and their families, and feelings of loneliness [8,9,10,11,12].

Between 40 and 70% of relatives of patients with major depression develop clinically significant anxiety and/or depressive symptoms [13,14], though these tend to recede as the severity of the depressive symptoms in their ill relatives reduce [14]. Additionally, family members often report a lack of adequate information on how to interact with their loved one [15], along with high levels of expressed emotions, in particular emotional overinvolvement and criticism [16].

The few available data about the impact of dysfunctional family environments and the long-term outcomes of MDD indicate that high levels of family burden, expressed emotions, and dysfunctional coping strategies by family members have a significant impact on the long-term outcomes of patients with major depression [17]. Heru et al. [13] and Keitner et al. [18] found that patients with MDD living in families with high levels of expressed emotion and burden achieve a full functional recovery after an acute episode less frequently than those living in less problematic families.

The available evidence shows that patients with MDD and their relatives should receive effective psychosocial interventions [19,20]. However, research on the best treatment options for MDD is scarce, and data are mainly derived from research on schizophrenia and bipolar disorder. For both of these disorders, psychoeducational family interventions (PFIs) have been shown to be particularly effective [21,22,23]. In fact, when provided to patients with schizophrenia and their family members, PFIs reduce relapses [24] and hospitalizations [25,26,27,28], and when provided to patients with bipolar disorder and their relatives, both during the acute phase [29] and in the long term [30,31], PFIs prevent relapses, reduce the number of acute episodes [32], improve patients’ adherence to pharmacological treatments [33,34], and foster psychosocial functioning [35] as well as coping strategies in their family members [36,37,38,39,40]. Based on this evidence, several international guidelines [41,42,43,44] recommend the use of PFIs in the clinical management of severe mental disorders, as well as the adoption of new delivery approaches which include modern technologies [38,39,40].

Despite the relevance of the family context to the recovery of patients with major depression [45,46], studies on the efficacy of PFIs in patients with major depression are limited. The efficacy of PFIs in patients with MDD and their relatives has been explored in only a few randomized controlled trials [47,48,49,50]. A recent metanalysis which included five RCTs found that the intervention had a small but significant effect in improving depressive symptoms [49], though the included studies had serious limitations. Thus, firm conclusions about the effectiveness of PFIs in the treatment of MDD cannot yet be drawn.

## 2. Aims

The primary aim of this study is to assess the efficacy of PFIs in a group of patients with a diagnosis of MDD in terms of: (1) reduction of depressive symptoms; (2) improvement of psychosocial functioning; and (3) increase of social contact. The efficacy of PFIs will also be evaluated in terms of: (1) reduction of relapses and hospitalizations at 18 months post-randomization; (2) improvement of adherence to pharmacological treatments and of patients’ quality of life; (3) reduction of self-stigmatization; (4) improvement of coping strategies and expressed emotion in family members, and reduction of family burden; (5) improvement of quality of life of family members. Finally, we will investigate whether the efficacy of the psychoeducational intervention is mediated by the presence of specific temperamental profiles and childhood traumas.

## 3. Methods

This will be a multicentric randomized controlled trial with blinded outcome assessments coordinated by the Department of Mental and Physical Health and Preventive Medicine at the University of Campania Luigi Vanvitelli, and it will be conducted in 24 Italian outpatient clinics.

Eligible patients and their families will be identified by their treating clinicians and referred to the specialized staff to be included in the study. After acceptance, an informed consent form will be signed and an appointment for baseline assessments will be made. Consent can be withdrawn at any time by both patients and their relatives. Patients will be randomly assigned in a 1:1 ratio to the experimental group or the control group. The randomization will be stratified according to the recruiting center and each patient’s age, sex, and educational level. The procedure will be implemented in the coordinating center using specific statistical software. The researchers and statisticians involved in the patients’ assessments will be blinded each patient’s allocation. The mental health professionals providing the interventions will not be blinded to the patients’ allocations.

Patients referred to the outpatient units of participating mental health centers will be invited to participate in the study if they meet the following inclusion criteria: (1) diagnosed with MDD according to the Diagnostic and Statistical Manual of Mental Disorders, Fifth Edition (DSM-5); (2) between 18 and 65 years of age; (3) admitted to the local mental health center for at least 6 months, with at least one access per month; (4) ability to provide written informed consent; (5) cohabitation with at least one family member.

Patients with intellectual disabilities will not be eligible, and neither will patients who experience a clinically relevant worsening of psychiatric symptoms (i.e., requiring a substantial change in the therapeutic dosage of psychiatric medications or access to emergency care or hospitalization) during the two months before recruitment. Patients with comorbid physical illnesses will not be excluded from the study unless the physical condition is so severe and disabling as to require intensive medical care. All recruited patients will continue to receive the treatments usually provided in their centers, which include regular outpatient psychiatric assessments, pharmacological treatments, and management of the side effects of medications. All patients will receive an adequate pharmacological treatment according to the NICE [51] guidelines for the whole duration of the study.

## 4. Interventions

The experimental intervention will consist of the Falloon psychoeducational family intervention, which was developed for the treatment of schizophrenia [52], but which we have adapted for the treatment of major depression. The experimental intervention will be administered individually to each recruited family and consist of the following six phases: (1) engagement of the family unit; (2) individual assessment; (3) family assessment; (4) informative sessions; (5) communication skills sessions; and (6) problem-solving skills sessions.

The informative sessions will deal with the following issues: (1) clinical characteristics of MDD, symptoms, prognosis, risk and protective factors; (2) available pharmacological and non-pharmacological treatments for MDD, indications, side effects and strategies to cope with them, treatment duration, risk factors for relapse, and effects related to abrupt cessation of the therapy; and (3) early warning signs. A specific informative session on suicide risk has been developed, and this will be administered only to patients who report suicidal ideation during the intervention or who have a history of suicide attempts or ideation.

The sessions will take place three times a month for a period ranging from 4 to 6 months (about 18 sessions in total). The number of sessions, as well as their frequency, may vary depending on each patient’s clinical situation. Sessions will have an average duration of 60–90 min and will require the active participation of all family members, including the patient. Between sessions, participants will be invited to organize “family meetings” to discuss the topics covered during the previous meeting, progress made, and problems encountered in implementing changes. Each meeting will ideally be divided into three phases: a first phase dedicated to clarifications and questions regarding the topics previously discussed, a second phase focused on the main topic of the meeting (the so-called “teaching phase”), and a final phase in which the key points addressed during the session are summarized and in which “homework” is assigned.

The control group will receive an educational five-session intervention, administered every 7–10 days, on the following topics: healthy lifestyle (diet and nutrition), stress management, regulation of circadian rhythms, and the management of medication side effects. The control group sessions will last 60–90 min and will be as interactive as possible. At the end of each session, participants will receive leaflets and cards summarizing the key-points addressed during the session.

Treatment fidelity among the different centers will be secured by the development of manuals for both interventions and by continuous supervision, either on site or via phone calls.

The interventions will be discontinued under the following circumstances: (1) if patients or their relatives are unable to attend more than five consecutive psychoeducational sessions; (2) if patients are hospitalized or experience any affective relapse during the intervention; or (3) if patients or relatives withdraw their consent.

## 5. Training of Mental Health Professionals

Two mental health professionals per center (at least one being a psychiatrist) will participate in a four-day training course on the use of the interventions. During the training, a manual explaining how to conduct the experimental intervention will be provided to participants, and several role-play sessions will be organized. An additional training course on the use of assessment instruments will be organized, and this will include the following phases: (a) presentation of the assessment instruments; (b) group reading; (c) role-play; and (d) video-recorded interviews. In each center, at least one researcher who is not involved in the administration of the interventions will be trained in the use of assessment tools and inter-rater reliability.

## 6. Ethical Issues

This study will be carried out in accordance with globally accepted standards of good practice and in agreement with the Declaration of Helsinki and with local regulations. The study investigators will ensure that all mental health professionals involved in the study are qualified and informed about the protocol, interventions, and trial-related duties.

A formal ethical approval has been obtained by the Coordinating Center’s Ethics Committee (approval number: prot. 0032758/i). Each participating center will submit the approval to their local ethics committees.

## 7. Assessment Time and Instruments

The following activities are planned for the first three months of the study: (1) the obtaining of approval from local ethics committees; (2) the training of mental health professionals in the interventions and in the use of evaluation tools; and (3) the implementation of the interventions. The recruitment of the patients and their relatives will take place between month 4 and month 12. The patients will be randomly assigned by the coordinating center only after the receipt of written informed consent. The implementation of the intervention will take place between month 3 and month 18. Assessments will be performed between month 3 and month 36. The timeline of the study is shown in Table 1.

The socio-demographic and clinical characteristics of the patients and their relatives will be collected at baseline through validated assessment instruments (Table 2). Researchers participating in the study will be blinded to patient allocation. All patients will be assessed at the following time points: baseline (T0); 6 months post-randomization (T1); 12 months post-randomization (T2); and 24 months post-randomization (T3). All data will be collected during paper and pencil interviews. Each assessment is expected to take about 90/120 min per patient and 60/90 min per relative. During the evaluation, if the participant feel distressed or exhausted, the assessment can be stopped and another appointment can be scheduled for the following 2/3 days.

## 8. Anticipated Results

### 8.1. Primary Outcomes

We expect that, at the end of the intervention, the MDD patients in the experimental group will show a significant reduction in the severity of their depressive symptoms compared with those in the control group. Moreover, we also expect the treated patients to report an improvement in their functioning and an increase in their social network at six months (i.e., improvements in the number of weekly social contacts, practical and emotional support from their social network, and an improvement in the quality of their intimate supportive relationships). We expect that these results will be maintained after 12 and 24 months, albeit with a reduction in magnitude.

### 8.2. Secondary Outcomes

We expect that, compared with those in the control group, the patients receiving the PFI will report a significantly lower number of relapses and hospitalizations after 12 and 24 months as well as improvements in their adherence to pharmacological treatments, quality of life (evaluated using the Manchester Short Assessment of Quality of Life), and coping strategies, and a reduction in their internalized stigma. With respect to coping strategies, we expect an increase in the relatives’ problem-oriented coping strategies (i.e., maintenance of social interests, involving the patient in social activities, positive communication with the patient, seeking information, talking with friends and/or relatives about the patient’s situation) and a reduction in emotion-focused coping strategies (i.e., collusion, avoidance, resignation, coercion, use of alcohol and/or drugs). Moreover, we expect reductions in objective and subjective family burden and improvements in expressed emotions and quality of life, both at the end of the intervention and at follow-ups.

### 8.3. Exploratory Outcomes

The presence of childhood traumas and certain affective temperaments (i.e., cyclothymic, irritable, and hyperthymic temperaments) can be considered possible predictors and/or mediators of response to psychosocial treatments. Thus, these risk factors will be included as possible moderators in order to assess the influence of these variables on the efficacy of the experimental intervention. Additionally, a series of clinical indicators of illness severity will be collected (including age at onset of the first episode, previous psychiatric hospitalizations, number and type of suicide attempts, alcohol and drug use, presence of psychotic symptoms during acute phases, severity of symptoms at baseline, and adherence to pharmacological treatments), and these will be used as explanative variables. Multivariable models will be corrected for these variables in order to identify patients who are more likely to be responsive to the effects of PFIs.

## 9. Statistical Analyses

### 9.1. Power Analysis

The sample size was estimated with respect to the measurement of the main outcomes of the study, and the measure that required the largest sample size (HAM-D) was taken as the reference. Specifically, a sample size sufficient to reduce the total HAM-D score by 10%, with a two-tailed probability of 0.05% and a power of 90%, was estimated. The multicenter design of the study required an adjustment of the sample size estimate in accordance with the value of the intracluster correlation coefficient, set at 0.030, and the coefficient of variation of cluster sizes, set at 0.400. The estimated sample size is 176 patients, 88 for each arm. Based on previous studies conducted with similar patient populations, a dropout rate of 15% is expected. Therefore, it will be necessary to recruit 384 families, 192 for each arm. Each participating center is expected to recruit 16 families (8 for each arm).

### 9.2. Statistical Analyses

The statistical analyses will be conducted in accordance with the intention-to-treat analysis. Missing data will be handled using a last observation carried forward (LOCF) analysis. Descriptive statistics will be calculated for the dependent and confounding variables for both groups. Confidence intervals will be calculated at 95%.

The analytical plan will include the following: (1) the use of generalized estimating equations (GEEs), which will provide an estimation of the impact of the intervention on the primary and secondary outcomes while controlling for multiple confounders (i.e., age, gender, illness duration, number of previous relapses, severity of symptoms at baseline, previous psychiatric admissions, physical and mental comorbidities, presence of psychotic symptoms during acute phases, suicidality, pharmacological treatments); (2) the application of a structural equation model to identify possible mediators and moderators of intervention efficacy (i.e., specific temperamental profiles, the presence of childhood trauma and/or abuse, severity of symptoms at baseline, the patient’s psychosocial functioning at baseline, number of family members, the relatives’ coping strategies and levels of expressed emotions at baseline); and (3) the use of a multivariate logistic regression model to identify predictors of a poor response to the experimental intervention.

## 10. Discussion

Although most of the relevant studies have been carried out with the families of patients with schizophrenia and bipolar disorder, evidence has shown that family functioning also has a significant impact on the course of MDD [67,68]. Relatives of patients with MDD report high levels of burden, including financial difficulties (which are mainly due to loss of productivity in both patients and caregivers), reduced social contact, problems with marital relationships, emotional exhaustion, and worries about the future. Family members also report high levels of anxiety and depressive symptoms, feelings of loneliness and hopelessness, and insomnia [47]. A direct correlation between family burden and patients’ adherence to treatments has been found, and this further emphasizes the importance of family members as allies in the treatment of patients with MDD.

The coping strategies of relatives are also significantly correlated with patient outcomes and with the mental health and wellbeing of relatives. In fact, the adoption of positive thinking and problem-oriented coping strategies by relatives is associated with a decrease in their levels of anxiety; in contrast, the adoption of avoidant coping strategies increases depressive and anxiety symptoms in caregivers, as well as anxiety in patients [15]. Surprisingly, few reports about family burden in MDD and its consequences for the medium- and long-term outcomes of patients are available. This can be explained by the fact that, in the past, major depression was thought to be a less burdensome mental disorder compared with schizophrenia, bipolar disorder, and substance abuse disorders [69].

It may seem obvious to suggest that interventions aimed at enhancing caregiver well-being and reducing caregiver distress may have a positive impact on patient outcomes [49]. In particular, among family interventions, psychoeducational family interventions are recognized as one of the most effective add-on treatments that can be used in conjunction with pharmacological therapy for people with bipolar disorder and schizophrenia [70,71,72,73], while there is little evidence concerning the efficacy of PFIs in the treatment of MDD. The few available studies report that PFIs contribute to shortened hospital admissions, reduced symptom severity, improved social functioning, and increased subjective well-being [74]. In addition, other studies suggest that psychoeducational family interventions may play a role in the prevention of suicidality and disease recurrence [51,74]. It has to be noted that the most recent meta-analysis of the efficacy of PFIs in patients with MDD [49] included only 301 patients with MDD from five randomized controlled trials. The authors reported that the studies had several limitations, including methodological weaknesses such as a lack of detail concerning the randomization process, a lack of blinding assessments, and low certainty concerning each outcome considered in the studies. Therefore, replications of these RCT results are desperately needed.

Our study will aim to overcome these limitations. In fact, one of the main novelties of the present study is the fact that the efficacy of PFIs will be tested on different clinical outcomes and will not be limited to depressive symptoms, i.e., rather than adhering to a recovery-oriented approach to mental disorders [75], the study will be more in line with the needs and values of patients [76,77,78]. It will also evaluate the efficacy of the experimental intervention on several psychosocial and psychological domains, both in patients and in their relatives. The majority of existing studies have assessed the efficacy of the intervention over the short and middle term, while data concerning its efficacy over a longer term are lacking. For this reason, we included a two-year follow-up assessment in order to verify whether the effects of the intervention, usually seen after 6 and 12 months, are maintained over a longer period. Moreover, a major strength of our trial is its adoption of a well-known and validated family psychoeducational model, which has been adapted for the treatment of patients with MDD. Specifically, the Falloon psychoeducational intervention was initially developed for the community management of schizophrenia [19], and then adapted for patients with bipolar disorder [79]. In these disorders, the efficacy of this model has been well documented in RCTs and in real-world trials. To our knowledge, its efficacy has never been tested in patients with MDD. Major depression may represent an ideal target for this approach since it is highly dependent on stressful life events and on family context [42].

Moreover, to our knowledge, no study has assessed the possible mediators and moderators of the efficacy of PFIs, and this is undoubtedly a novel aspect of the present study. Specifically, in our study, we tested the hypothesis that affective temperaments and childhood traumas can impact the responsiveness of patients to family treatments. It has been reported that affective temperaments play a significant role in influencing the long-term outcomes of affective disorders, and that cyclothymic and irritable affective dispositions are associated with a reduced response to pharmacological and/or psychosocial interventions [80]. Regarding childhood traumas, several studies have reported that the presence of physical and psychological abuse, as well as the presence of traumas during childhood, are associated with worse outcomes in MDD patients, reduced problem-solving and social skills, increased disease burden, and increased risk of suicidality [81]. Patients with childhood traumas may present more severe symptoms, be more reluctant to implement changes proposed during an intervention, and show resistance to pharmacological and psychosocial treatments.

Another novelty of the present study is its multicentric nature. In fact, the 24 centers involved in the study are highly representative of the whole Italian nation. The sample size is very large (a total of 384 families enrolled). To our knowledge, no study so far carried out on the efficacy of PFIs in MDD patients and their relatives has been as representative of an entire European country.

Additionally, in the present trial, assessments will be blinded, meaning that the researchers and statisticians will be unaware of the allocations of the patients and their relatives. We decided to collect a variety of “hard clinical indicators”, including age at onset of mental illness, previous hospitalizations for mental illness, suicide attempts, and alcohol and drug use, all of which will be used as explanative variables. These variables will be used to assess potential moderators of the efficacy of PFIs and will inform us about factors that can potentially mitigate the efficacy of such interventions [82,83].

The clinical breakthrough power of this study consists of several aspects. First, the emphasis is not only on reducing symptom severity, but also on restoring a meaningful life so that patients feel satisfied with their personal and social lives [84]. Subjective feelings of being supported by relatives and friends strongly influence the recovery of patients. In fact, the available data show that 51.2% of patients with major depression consider lack of social support an important complication hindering their recovery [85].

Another innovative aspect of our proposed study is the total duration of the intervention (from 4 to 6 months), which includes an average of 18 planned sessions. In addition, the experimental intervention will be flexible, meaning that the number of sessions and their durations can be modulated according to the specific needs of each family. This is intended to increase the scalability of the intervention under routine conditions.

However, the proposed study has some unavoidable limitations. First, some psychopathological dimensions will be assessed using self-assessment instruments. This is necessary if we want to apply the intervention in real-world settings, where the resources are limited, and long evaluations are very difficult. Second, the total number of delivered sessions will not be standardized. Although this might seem a limitation, according to the original PFI Falloon model, psychoeducation should be adapted to the needs of families and, thus, it cannot be too structured. However, a minimum number of 12 sessions will be delivered in order to overcome this limitation. Moreover, we will not include an assessment of the barriers hindering the implementation of PFIs in the participating centers. This is because the main focus of our study is to explore the efficacy of PFIs for patients with MDD and their family members; the scalability of the intervention can be assessed in a forthcoming study, after its efficacy has been documented. Lastly, another possible limitation is the shorter duration of the intervention provided to the control group. However, most RCTs involving psychosocial interventions explore the efficacy of an experimental intervention against treatment as usual, or against a waiting list (meaning no active comparator), or against no intervention. Thus, we believe that the comparison between two active interventions adds value to our protocol.

## 11. Conclusions

In conclusion, from a clinical perspective, the proposed study has the potential to provide valuable insight into the efficacy and effectiveness of family psychoeducation interventions for patients with MDD and their families. If the intervention proves to be effective in reducing depressive symptoms, improving psychosocial functioning, and reducing relapse rates, it will have to be added to the clinical management of MDD, according to the personalized treatment approach.

## Figures and Tables

**Table 1:** Timeline of the project.

Month	1	2	3	4	5	6	7	8	9	10	11	12–18	19–36
**Obtaining approval of ethical committees**													
**Training in the use of assessment instrument**													
**Training in the use of interventions**													
**Recruitment of patients and their relatives**													
**Administration of interventions**													
**Follow-up assessments**													

**Table 2:** Assessments instruments.

Assessment Tool	Assessed Domains	Description	Study Population	Assessment Time-Points
**Patients’ socio-demographic schedule**	**Patients’ socio-demographic and clinical characteristics**	**Recorded information will include illness duration, age at onset, number of affective episodes and previous hospitalizations, age at first hospitalization, number of suicide attempts, age, gender, educational level, occupational status, and pharmacological and psychosocial interventions.** **The information will be compiled by the researcher in collaboration with the patient. If the information is inadequate, or if the researcher is not sure about the patient’s reliability, other sources (e.g., treating physician, relatives) can be consulted.**	**Patients**	**T0, T1, T2, and T3**
**Relatives’ socio-demographic schedule**	**Relatives’ sociodemographic characteristics**	**Recorded information will include age, gender, educational level, occupational status, nature of relationship with the patient, number of years cohabiting with the patient, and mean number of daily hours spent with the patient.**	**Relatives**	**T0**
Hamilton Depression Rating Scale (HAM-D) [53]	Severity of depressive symptoms	The HAM-D includes 17 items. Of these, eight items are scored from 0 (absent) to 4 (severe), while nine are scored from 0 to 2. The total score is the sum of the item scores, and ranges from 0 to 52 points.	Patients	T0, T1, T2, and T3
Personal and Social Performance Scale (PSP) [54]	Psychosocial functioning	The total score (which can range from 0 to 100) can be used to assess the patient’s overall functioning (higher scores indicate higher functioning). Ratings are based mainly on assessments of a patient’s functioning in four main areas: (1) socially useful activities; (2) personal and social relationships; (3) self-care; and (4) disturbing and aggressive behaviors.	Patients	T0, T1, T2, and T3
Family Problems Questionnaire—Patients’ and relatives’ versions (FPQ) [55]	Practical and psychological burden	The FPQ is a self-administered questionnaire containing 34 items. These items are rated on a 4-point scale, from 1 (never) to 4 (always).	Patients and relatives	T0, T1, T2, and T3
**Social Network Questionnaire (SNQ) [56]**	**Social contacts**	**The SNQ is a self-administered questionnaire which includes 15 items grouped into 4 subscales (practical support, affective support, social and professional help, and help in an emergency). Items range from 1 (never) to 4 (always).**	**Patients and relatives**	**T0, T1, T2, and T3**
Hamilton Anxiety Rating Scale (HAM-A) [57]	Severity of anxiety symptoms	The HAM-A is a 14-item questionnaire developed to measure the severity of anxiety symptoms, both psychic (mental agitation and psychological distress) and somatic (physical complaints related to anxiety). The score for each item ranges from 0 (not present) to 4 (extreme severity).	Patients	T0, T1, T2, and T3
Morisky Medication Adherence Scale [58]	Adherence to pharmacological treatments	This is a 4-item questionnaire. The response options are yes/no for each item, and a five-point Likert scale is used for the last item.	Patients	T0, T1, T2, and T3
Brief Temperament Evaluation of Memphis, Pisa, Paris, and San Diego (B-TEMPS-M) [59]	Affective temperaments	The B-TEMPS-M includes 35 items and uses a five-point Likert scale.	Patients	T0
Childhood Trauma Questionnaire (CTQ) [60]	Childhood trauma and abuse	The CTQ is a 70-item questionnaire which uses a five-point Likert scale. Ratings are grouped into five subscales: emotional abuse, physical abuse, sexual abuse, physical neglect, and emotional neglect.	Patients	T0
Clinical Global Impression Scale (CGI) [61]	Illness severity (CGI-S), global changes in the severity of symptoms (CGI-C), and therapeutic response.	The CGI-S employs a seven-point scale, from 1 (normal) to 7 (the most severely ill patients). CGI-C scores range from 1 (very much improved) to 7 (very much worse). Treatment response ratings should take into account both the therapeutic efficacy and the treatment-related adverse events, and they range from 0 (marked improvement and no side effects) to 4 (unchanged or worse, and the side effects outweigh the therapeutic effects).	Patients	T0, T1, T2, and T3
Columbia Suicide Severity Rating Scale (C-SSRS) [62]	Suicidality	The clinician-administered version of the C-SSRS (screening version) will be administered. An individual’s suicidal ideation is rated on a scale from 1 (wish to be dead) to 5 (active suicidal ideation with a specific plan and intent). An individual’s previous suicide attempts are rated on a scale from 1 (actual attempt, defined as “a potentially self-injurious act committed with at least some wish to die, as a result of act”) to 4 (preparatory acts or behavior, defined as “Acts or preparation towards imminently making a suicide attempt”). All patients are rated as positive for suicide attempts if they respond positively to one of the four items assessing suicidal behaviors.	Patients	T0, T1, T2, and T3
Level of Expressed Emotion Scale (LEE) [63]	Expressed emotions	The LEE is a 60-item self-administered assessment instrument. The items are rated as true or false and are grouped into four subscales: (1) intrusiveness; (2) emotional response; (3) attitude toward illness; and (4) tolerance/expectations.	Patients and relatives	T0, T1, T2, and T3
Insomnia Severity Index (ISI) [64]	Insomnia	The ISI is a seven-item questionnaire. Each item is rated on using a five-point Likert scale. It is a brief test that assesses the different components of insomnia.	Patients	T0, T1, T2, and T3
Manchester Short Assessment of Quality of Life for the evaluation of quality of life (MANSA) [65]	Quality of life	The MANSA is a 17-item questionnaire, 12 of which are rated using a 7-point Likert scale, ranging from 1 (“could not be worse”) to 7 (“could not be better”), and 5 of which are rated as either yes or no.	Patients and relatives	T0, T1, T2, and T3
Internalized Stigma of Mental Illness Inventory (ISMI) [66]	Stigma	The ISMI is a 29-items questionnaire for evaluating the subjective experience of stigma.	Patients	T0, T1, T2, and T3
**Family Coping Questionnaire (FCQ) [56]**	**Coping strategies**	**The FCQ is a self-administered questionnaire consisting of 34 items rated using a 4-point scale, from 1 (never) to 4 (always). The items can be grouped into the following 11 subscales: seeking information on a patient’s illness, positive communication toward the patient, relatives’ maintenance of social interests, patient’s involvement in social activities, talking with friends about the patient’s condition, coercion, avoidance, resignation, use of alcohol and drugs, collusion, and searching for spiritual help. A higher score is indicative of a stronger endorsement of each coping strategy.**	**Relatives**	**T0, T1, T2, and T3**

## Data Availability

Not applicable.
